# Performance of the Artificial Intelligence large language models ChatGPT 3.5, Gemini (Google Bard), ChatGPT 4.0, and Gemini 2.5 flash in surgical subspecialty questions of Brazilian medical residency exams

**DOI:** 10.31744/einstein_journal/2026AO1436

**Published:** 2026-01-28

**Authors:** Maria Clara Pimenta de Figueiredo, Victor Hugo Alves Diniz, Ana Clara de Campos Granado, Gabriel Chagas Lutfala Paulino, Gabryella Rodrigues de Oliveira

**Affiliations:** 1 Universidade Estadual de Campinas Campinas SP Brazil Universidade Estadual de Campinas, Campinas, SP, Brazil.

**Keywords:** Large language models, ChatGPT, Generative artificial intelligence, Gemini, Artificial intelligence, Education, medical

## Abstract

**Objective:**

Given the rapid advancement of Artificial Intelligence and its significant impact on medical education, particularly with the development of large language models, such as ChatGPT and Gemini, ChatBots have shown an increasing ability to support clinical reasoning. Regarding this, there has been a growing interest in assessing the performance of ChatBots in medical examinations. However, there are insufficient data on these tools for addressing Brazilian medical exam-related inquiries, as well as their potential as educational tools for medical students. Therefore, we aimed to evaluate the performance of Artificial Intelligence on residency entrance exams for surgical subspecialties in six different surgical residency programs.

**Methods:**

We analyzed the performance of ChatGPT 3.5, Gemini (Google Bard), ChatGPT 4.0, and Gemini 2.5 Flash on 464 practice questions from six major institutions in São Paulo that offer surgical medical residency programs. The questions were multiple-choice, and each had a single correct answer.

**Results:**

Overall, ChatGPT 3.5 correctly answered 257 (55.4%), Gemini (Bard) 237 (51.1%), ChatGPT4.0 360 (77.6%), and Gemini 2.5 Flash 376 (81%) out of 464 questions, showing a substantial increase in performance.

**Conclusion:**

These findings underscore the potential of advanced large language models to support medical education. Although it is unlikely that these platforms will replace the clinical decision-making skills of surgeons trained by higher education institutions, when used appropriately, they may serve as an adjunct tool for medical education.

## In Brief

The application of Artificial Intelligence has been expanded to medicine and presents a promising future for medical education. Despite technological advances, it is still important to consider the role of professionals in developing the essential clinical judgments required for medical practice.

## Highlights

■ ChatGPT and Gemini are showing increased ability to accurately answer multiple-choice questions on medical exams.
■ There was no statistical significance in the rate of correct answers by ChatGPT 3.5 and Gemini 1.5. However, we observed that ChatGPT 4.0 performed significantly better, and so did Gemini 2.5 Flash, when comparing to the literature.
■ The question taxonomy did not appear to be a relevant factor regarding the success rate of the models.

## Graphical abstract

**Figure:**
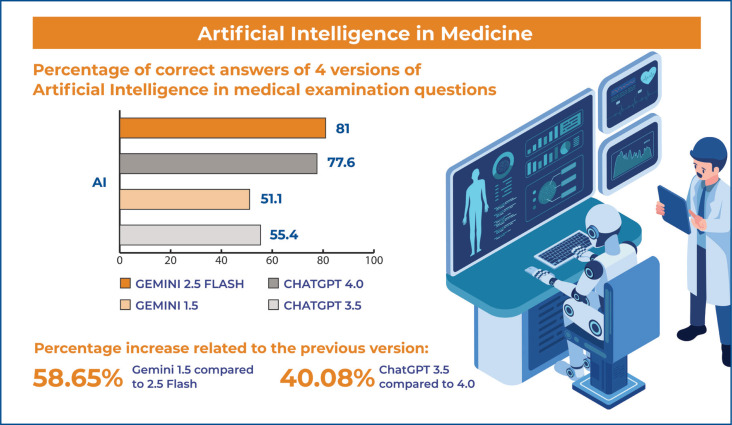

## INTRODUCTION

Alan Turing is one of the founders of modern computers and Artificial Intelligence (AI). The Turing test is based on the fact that the intelligent behavior of a computer depends on its ability to achieve human-level performance in tasks related to cognition.^(1)^ Therefore, AI is a technique for creating intelligent machines^(2)^ which have the ability to imitate cognitive tasks, such as image and speech recognition, by recognizing patterns and making accurate predictions.^(3)^

The AI methodology is based on statistical data analysis or machine learning. Machine learning allows software algorithms to be designed for desired applications. When this system is fed with information from a database, it identifies patterns, enabling the machine to "learn" and apply this knowledge to similar future scenarios. Machine learning excels in automatically learning and extracting relevant information from raw data and making decisions on its own, similar to the human brain.^(2)^

The foundation of evidence-based medicine is the establishment of clinical correlations by developing associations and patterns using existing information database.^(1)^ Recently, applications of AI in medicine have been expanded,^(4)^ being used to analyze clinical, behavioral, and environmental information; applying them to algorithms capable of recognizing the appearance of certain symptom groups or specific clinical and radiological images by utilizing learning and pattern recognition principles;^(1)^ and therefore improving evidence-based decisions. In this context, diagnoses and therapies can now be accessed by doctors and patients with just a few clicks.^(5)^

The accuracy of AI algorithms as diagnostic methods has been investigated. McKinney et al.^(6)^ showed that a trained AI system outperformed trained radiologists in predicting breast cancer using mammography images, with an absolute margin of 11.5%. Liu et al.^(7)^ demonstrated that another AI system identified 26 dermatological conditions (representing 80% of dermatology complaints in primary care), with a performance similar to that of trained dermatologists and superior to that of primary care clinicians and nurses.

Based on these drastic changes in medical practice and decision-making process, in 2019, the Standing Committee of European Doctors (SCED) emphasized the importance of using AI in medical education by proposing that AI systems should be integrated into medical education and residency training.^(6)^Approximately 85% of medical students believe that AI facilitates healthcare professionals’ access to health information and 70% believe that this use can help reduce medical errors, while only 35% believe that they are capable of assessing the reliability of diagnostic information provided by AI.^(5)^ Other studies have reported that medical students have shown increasing interest regarding the revision of medical curricula to adapt to the AI-influenced healthcare environment, indicating that trainee doctors are taking on new roles in the face of the challenges posed by these new diagnostic systems.^(8)^

ChatBots are AI systems programmed to understand, process, and generate human language. They are trained on input data to respond to a wide range of queries and retrieve information from the Internet using advanced language processing models.^(8)^ The emergence of ChatBots, such as ChatGPT and Gemini, has changed the landscape of education and access to knowledge.^(9)^ These platforms have great ability to synthesize information to generate precise answers and have been used as aid tools in the training of medical professionals.^(9)^

Currently, in Brazil, Medical Residency is a postgraduate education modality for doctors, characterized as a specialized course through in-service training. The National Medical Residency Commission regulates and supervises Residency programs.^(10)^ It also regulates, supervises, and evaluates institutions offering medical residencies,^(11)^ ensuring that the Medical Residency Program fulfills its role in the formation and improvement of medical practice. Multiple-choice questions are considered popular for testing applied knowledge^(12)^ and are the chosen modality of entrance examinations for surgical subspecialty residency in Brazil.

As AI-based technologies are continuously evolving, some studies have suggested that advances in natural language processing and large language models may offer potential solutions in healthcare education, which could be pivotal in automating the use of these tools for multiple-choice questions.^(12)^ In this context, studies have shown increasing interest in assessing the performance of ChatBots in medical examinations. However, at present, there are insufficient data on ChatGPT and Gemini in addressing Brazilian surgery-related exam questions as well as their potential as educational tools for Brazilian doctors in training.

## OBJETIVE

The aim of this study was to evaluate the performance of Artificial Intelligence chatbots (ChatGPT-3.5, ChatGPT-4.0, Gemini, and Gemini 2.5 Flash) in surgical subspecialty residency entrance exam questions from the most competitive programs in São Paulo and to analyze their potential advantages and limitations in surgical education and decision-making training.

## METHODS

ChatGPT3.5 and ChatGPT 4.0 (OpenAI) were accessed through ChatGPT's website, and Bard and Gemini 2.5 Flash (Google LLC) were also accessed thought their webpages.

Five hundred and eighty multiple-choice questions (from 2024) from the exam question bank of six different surgical subspecialty residency programs were selected. Two hundred and twenty of these questions were from the *Universidade de São Paulo* (USP - SP), eighty were from the *Universidade Estadual de Campinas* (UNICAMP), fifty from the *Universidade Federal de São Paulo* (UNIFESP), eighty from the *Instituto de Assistência Médica ao Servidor Público Estadual de São Paulo* (IAMSPE - SP), fifty from the *Sistema Único de Saúde do Estado de São Paulo* (SUS - SP), and one hundred from the *Universidade de São Paulo Campus de Ribeirão Preto* (USP-RP). These institutions were selected because they are highly preferred for admission.

Of the 580 practice questions, questions that required the interpretation of images and radiological findings were excluded as the figures could not be interpreted by these large language models without any bias. Thus, four hundred and sixty-four questions were used to test the AI platforms, and each question had a single correct answer. We chose ChatGPT and Gemini because they are both freely available on the Internet, are the most studied,^(9,13,14)^ and we were able to compare the previous and updated versions of both ChatBots.

We allocated all questions into six categories in terms of the originating institution and into four categories based on the taxonomy of each question: (i) conceptual questions: questions requiring recall of anatomical concepts, epidemiology, description of surgical techniques, indications, and contraindications of procedures; (ii) diagnosis: questions addressing a medical case and requiring clinical reasoning to reach a diagnostic hypothesis; (iii) conduct and management: questions addressing a medical case and testing the decision-making process; and (iv) diagnosis and conduct. Each question was copied with its answer choices and pasted into ChatGPT 3.5 and Gemini (Bard), and then into ChatGPT 4.0 and Gemini 2.5 Flash. We refreshed the webpage for every new question entry to avoid bias. A χ^2^-test was performed to compare the performance of both AI platforms and the performance of the updated ChatBots: ChatGPT 4.0, available for free since April 2024, and Gemini 2.5 Flash, available since June 2025. In addition, a *t*-test was performed to compare the percentage of correct answers obtained herein with the overall average of correct answers analyzed in other similar studies. Statistical analyses were performed using IBM SPSS Statistics (version 2.0.0.0.0).

## RESULTS

On average, ChatGPT3.5 correctly answered 257 (55.4%), whereas Gemini 1.5 correctly answered 237 (51.1%) of the 464 questions. ChatGPT 3.5 performed better than Gemini (Bard) across all six surgical subspecialty residency program entrance exams. When using updated versions of these ChatBots, we observed that ChatGPT 4.0 correctly answered 360 (77.6%) and Gemini 2.5 Flash correctly answered 376 (81%) of the 464 questions, showing a substantial increase in performance (Table 1). In this second analysis, we did not observe superior performance of ChatGPT 4.0 over Gemini 2.5 Flash across all exams. We allocated the questions into six categories, where each category represents a single institution used as a database.

**Table 1:** Number of questions per institution and correct answers per Artificial Intelligence

Institution	Questions (n)	ChatGPT 3.5 (%)	Gemini (Bard) (%)	ChatGPT 4.0 (%)	Gemini 2.5 Flash (%)
USP - SP	158	94 (59.5)	86 (54.4)	132 (83.5)	136 (86.1)
UNICAMP	65	36 (55.4)	35 (53.8)	52 (80)	55 (84.6)
UNIFESP	46	23 (50)	18 (39.1)	24 (52.2)	31 (67.4)
SUS-SP	46	19 (41.3)	19 (41.3)	30 (65.2)	29 (63)
IAMSPE - SP	67	38 (56.7)	38 (56.7)	52 (77.6)	54 (80.6)
USP-RP	82	47 (57.3)	41 (50)	70 (85.4)	71 (86.6)
Total	464	257 (55.4)	237 (51.1)	360 (77.6)	376 (81)

We performed a one-sample K-S test, which demonstrated differences in correct answer rates in the separate ChatBot analysis. We found that ChatGPT 3.5, Gemini (Bard), ChatGPT 4.0, and Gemini 2.5 Flash had similar performances across the six categories evaluated, with p values of 0.138, 0.398, 0.167, and 0.223, respectively. ChatGPT 3.5 performed the best in USP-SP (59.5%) and worst in SUS-SP (41.3%); Gemini (Bard) performed the best in IAMSPE-SP (56.7%) and worst in UNIFESP (39.1%); and ChatGPT 4.0 and Gemini 2.5 Flash performed the best in USP-RP (85.4% and 86.6%, respectively). While ChatGPT 4.0 was the worst in UNIFESP 52.2%, Gemini 2.5 Flash performed the worst in SUS-SP (63%). The level of association between the ability of ChatGPT 3.5 and Gemini (Bard) to correctly answer the same question was 34.5% (p<0.001), while that between ChatGPT 4.0 and Gemini 2.5 Flash was 46.5% (p<0.001).

When comparing ChatGPT 3.5 and ChatGPT 4.0 (Table 2) using the χ^2^-test, we observed a statistical increase in overall performance (p<0.001). The increase was however not homogeneous across the six categories because we did not observe a significant difference in performance between ChatGPT 3.5 and ChatGPT 4.0 in terms of UNIFESP exam questions (p=0.768). Gemini 2.5 Flash showed a similar increase in performance compared to Gemini (Bard), presenting a statistically higher rate of correct answers across almost all categories, except for SUS-SP (p=0.077) (Table 3).

**Table 2:** Comparison of number of questions per institution and correct answers by ChatGPT versions 3.5 and 4.0

Institution	Questions (n)	ChatGPT 3.5 (%)	ChatGPT 4.0 (%)	p value
USP - SP	158	94 (59.5)	132 (83.5)	<0.001
UNICAMP	65	36 (55.4)	52 (80)	<0.001
UNIFESP	46	23 (50)	24 (52.2)	0.768
SUS-SP	46	19 (41.3)	30 (65.2)	0.039
IAMSPE-SP	67	38 (56.7)	52 (77.6)	<0.001
USP-RP	82	47 (57.3)	70 (85.4)	<0.001
Total	464	257 (55.4)	360 (77.6)	<0.001

**Table 3:** Comparison of the number of questions per institution and correct answers by Gemini versions Google Bard and 2.5 Flash

Institution	Questions (n)	Gemini (Bard) (%)	Gemini 2.5 Flash (%)	p value
USP-SP	158	86 (54.4)	136 (86.1)	<0.001
UNICAMP	65	35 (53.8)	55 (84.6)	<0.001
UNIFESP	46	18 (39.1)	31 (67.4)	0.018
SUS-SP	46	19 (41.3)	29 (63)	0.077
IAMSPE-SP	67	38 (56.7)	54 (80.6)	<0.001
USP-RP	82	41 (50)	71 (86.6)	<0.001
Total	464	237 (51.1)	376 (81)	<0.001

For question taxonomy, we excluded questions from USP-SP that were specific to each surgical subspecialty and selected those applicable to all candidates. Thus, 375 questions were included in the final analysis. In our database, we identified 163 (43.5%) questions on conceptual knowledge, 33 (8.8%) on diagnosis, 146 (38.9%) on conduct and management, and 33 (8.8%) on diagnosis and conduct. We also observed that most questions from USP-SP, UNIFESP, and USP-RP were conduct-based questions (47.8, 63, and 53.7%, respectively) and those from UNICAMP, SUS-SP, and IAMSPE were conceptual questions (47.7, 65.2, and 76.1%, respectively). Differences in scores were not statistically significant (p>0.05) for question taxonomy for each AI model (Table 4). ChatGPT 3.5 and Gemini (Bard) both performed best in conceptual questions, while ChatGPT 4.0 and Gemini 2.5 Flash both performed best in the diagnosis category.

**Table 4:** Number of questions per taxonomy and correct responses by ChatGPT and Gemini

	Concept (n, %)	Diagnosis (n, %)	Conduct (n, %)	Diagnosis and Conduct (n, %)	p value
ChatGPT 3,5	87 (53.7)	14 (42.4)	78 (53.4)	20 (60.6)	0.519
Gemini (Bard)	92 (56.4)	15 (45.5)	69 (47.3)	12 (36.4)	0.118
ChatGPT 4.0	128 (78.5)	28 (84.8)	104 (71.2)	25 (75.6)	0.283
Gemini 2.5 Flash	125 (76.7)	30 (90.9)	117 (80.1)	27 (81.8)	0.309

## DISCUSSION

Previous studies have examined the performance of large language models in board examination questions, observing a performance rate of 46.3% in orthopedics,^(15)^ 56% in dermatology,^(16)^ and 62.4% in neurosurgery.^(13)^

When using the average number of correct answers presented in the literature (54.9%), we observed no statistically significant difference in the rate of correct answers by ChatGPT 3.5 and Gemini 1.5 (p=0.725 and p=0.112, respectively) when using *t*-test, the test value of which was the average number of correct answers predicted in the literature. However, we observed that ChatGPT 4.0 performed significantly better (p=0.015) and so did Gemini 2.5 Flash (p = 0.003) when comparing to findings in the literature.

Our findings support that there is a new era of technological breakthrough and that medical education is not lagging in this regard. Artificial large language models, such as ChatGPT and Gemini, are progressively exhibiting increased ability to generate responses to written prompts and accurately answer multiple-choice questions.^17^ Multiple studies have shown an optimistic perspective regarding the ability of these large language models to help students practice and improve their knowledge. However, AI models do have limitations, which include a potential bias in their sources,^(17)^ and we observed that the way medical issues are addressed by the enunciations of each multiple-choice question might influence an AI model's performance, although question taxonomy did not appear to be a relevant issue regarding the success rate of the models.

With the rapid evolution of newer generations of large language models, ChatGPT 3.5 and Gemini (Bard) became obsolete at the end of this study. However, we observed that the integration of AI models, such as ChatGPT 4.0 and Gemini 2.5 Flash, into medical education has shown promising advancements, particularly with the emergence of newer-generation models that demonstrate significant improvements in performance in terms of clinical reasoning, medical knowledge, and problem-solving skills with diagnostic accuracy.

## CONCLUSION

Although Artificial Intelligence models do not have the holistic knowledge and clinical experience of surgeons under training who are constantly exercising their ability to diagnose and manage clinical situations, which are imperative to the decision-making processes of a medical doctor, large language models show promise for future medical education. Newer-generation models rival the average human in terms of performance on standardized medical examinations. However, despite these advancements, we highlight the potential bias in these platform databases and draw attention to the need for human-led investigations into their reliability, interpretability, and impact on outcomes and decision-making processes. Nowadays, students worldwide are changing traditional study methods to ChatBots, but it is unlikely that these platforms will replace the nuanced diagnostic ability of surgeons trained by higher educational institutions in Brazil. It is important to consider the new roles of doctors and soon-to-be health professionals in this new scenario to develop the essential clinical judgments required in medical practice.

## Data Availability

The underlying content is contained within the manuscript.
